# The stem cell revolution: on the role of CD164 as a human stem cell marker

**DOI:** 10.1038/s41536-021-00143-1

**Published:** 2021-06-08

**Authors:** Suzanne M. Watt, Hans-Jörg Bühring, Paul J. Simmons, Andrew W. C. Zannettino

**Affiliations:** 1 Stem Cell Research, Nuffield Division of Clinical Laboratory Sciences, Radcliffe Department of Medicine, University of Oxford, Oxford, UK; 2 Myeloma Research Laboratory, Adelaide Medical School, Faculty of Health and Medical Sciences, University of Adelaide, Adelaide, Australia; 3 Cancer Program, Precision Medicine Theme, South Australian Health and Medical Research Institute, Adelaide, Australia; 4 Department of Internal Medicine II, Division of Hematology, Immunology, and Oncology, University Clinic of Tübingen, Eberhard Karls University, Tübingen, Germany; 5 Mesoblast Ltd., Melbourne, Australia; 6 Central Adelaide Local Health Network, Adelaide, Australia

**Keywords:** Regeneration, Translational research, Regenerative medicine

## Abstract

Accurately defining hierarchical relationships between human stem cells and their progeny, and using this knowledge for new cellular therapies, will undoubtedly lead to further successful treatments for life threatening and chronic diseases, which represent substantial burdens on patient quality of life and to healthcare systems globally. Clinical translation relies in part on appropriate biomarker, in vitro manipulation and transplantation strategies. CD164 has recently been cited as an important biomarker for enriching both human haematopoietic and skeletal stem cells, yet a thorough description of extant human CD164 monoclonal antibody (Mab) characteristics, which are critical for identifying and purifying these stem cells, was not discussed in these articles. Here, we highlight earlier but crucial research describing these relevant characteristics, including the differing human CD164 Mab avidities and their binding sites on the human CD164 sialomucin, which importantly may affect subsequent stem cell function and fate.

## Discovery of human CD164 as a haematopoietic and skeletal stem cell biomarker

Just over two decades ago, human (h) CD164 was identified as a functional biomarker on human haematopoietic precursors and their associated bone marrow stromal cells^1–3^. Importantly, a 1998 special focus report in ‘Blood’^3,4^ highlighted the findings that hCD164 was a sialomucin involved in human haematopoietic progenitor-stromal interactions, and that engagement of specific glycosylated hCD164 epitopes on quiescent human CD34+ haematopoietic stem/progenitor cells (HSPCs) could prevent their recruitment into the cell cycle^3,5^. Two decades on, by analysing human bone marrow Lineage (Lin)^neg^, CD34^+^, CD34^low^, and CD34^neg^ HSPCs with single cell RNAseq and improved surrogate transplantation models, Pellin and colleagues, in their article ‘A comprehensive single cell transcriptional landscape of human hematopoietic progenitors’ identified hCD164 as an important, reliable biomarker for defining the earliest branch points of hHSPC specification, which incorporates the basophil lineage and shows close similarities to murine lineage specification^6^. Interestingly, just prior to this observation, Chan and colleagues, in their paper entitled ‘Identification of the human skeletal stem cell’ meticulously purified, from hypertrophic zones of the growth plate in long bones, a subset of human PDPN^+^CD73^+^CD164^+^ skeletal stem cells (hSSC), which were negative for CD45, CD146, CD235, Tie2 and CD31, and at the apex of a hierarchy of stem cells that could transition into an early bone-cartilage-stromal progenitor^7,8^. These cells were demonstrated to meet the rigorous definition of hSSCs^9,10^, as being locally restricted to the bone, with the ability to clonally self-renew, differentiate into multiple lineages and reconstitute a haematopoietic microenvironment both in vitro and in vivo in surrogate serial transplantation models^7,8,11^. Importantly, and reminiscent of initial studies^3,4^, regulatory cross-talk was shown to exist between CD164^+^ hHSCs and these CD164^+^ hSSCs^7,8,11^.

While it is gratifying to see hCD164 identified as a potentially improved biomarker for hHSPC and hSSC isolation, we wish to highlight a critical issue not addressed in the three recent manuscripts cited above^3–5^, namely the choice of hCD164 antibody used for these purposes. As described below, at least three epitopes defined by three distinct Classes of hCD164 antibodies (Mabs) exist on hCD164 and representative of each class do not stain cells equivalently.

## The structure of the human CD164 biomarker

**Fig. 1:**
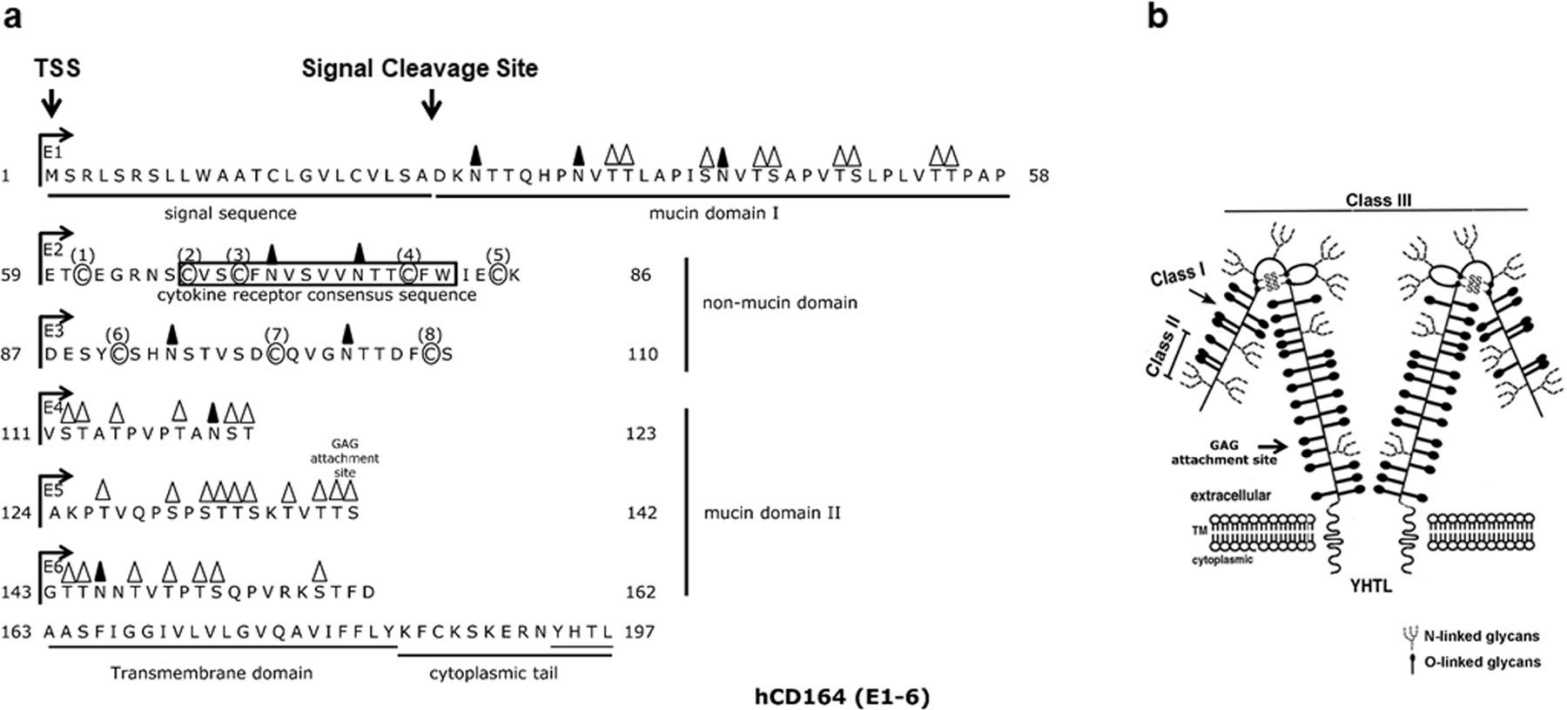
hCD164 structure, epitopes and splice variants. The *hCD164* gene is located on chromosome 6q21, comprises six exons (E1–6) and encodes a type 1 integral transmembrane sialomucin. **a** hCD164(E1–6) amino acid sequence, with exons (E), glycosylation, mucin domains and motifs. TM transmembrane region. The hCD164(E1–6) isoform with 9 N-linked and 32 O-linked glycans, and a glycosaminoglycan (GAG) attachment site at the end of E5 and beginning of E6. The first mucin domain is encoded by E1, the cysteine rich non-mucin domain by E2 and E3, the second mucin domain by E4 to part of E6, and the transmembrane region, cytoplasmic domain and 3′UTR by the remainder of E6. A cytokine binding pocket is predicted to lie in the non-mucin domain. **b** Diagrammatic representation of hCD164, indicating regions where the Class I, II, and III hCD164 Mabs bind, and putative intra-molecular disulphide bridges. The molecular mass of the hCD164 monomer or homodimer varies from 80–100 kD to 160–180 kD under respective non-reducing and reducing conditions, while the molecular mass of the GAG modified hCD164 or the hCD164 tetramer exceeds 220 kD. Epitope recognition sites are also shown for representatives of each Class of CD164 Mabs and further elaborated in Fig. 2.

*hCD164* was cloned from a bone marrow stromal cDNA expression library using two Mabs, 103B2/9E10 and 105A5^3,5^, and was demonstrated to be partially homologous to the *MGC-24* cDNA^5,12^. The *hCD164* gene spans at least 22 kb of genomic DNA, is located on chromosome 6q21, comprises six exons (E1–6) interspersed with five introns, and encodes a sialomucin^2,3,5,13–15^. Its extracellular domain comprises two highly O-glycosylated mucin domains interrupted by a cysteine-rich non-mucin domain^3,5,13,14,16,17^. Four splice variants exist, three involving differential splicing of complete exons and one involving splicing in the 3′ UTR^3,5,13,14,16,17^. The former comprises hCD164(E1–6) containing all six exon-encoded domains, hCD164(EΔ5) lacking the exon 5-encoded domain and hCD164(EΔ4) lacking the exon-4-encoded domain, and comprising the respective 197, 178, and 184 amino acid polypeptides (Fig. 1). The major isoform hCD164(E1–6), and hCD164(EΔ5) are present on both hHSPCs and human mesenchymal stem-stromal/skeletal stem cells (hMSC/hSSC)^1–8,13–31^.

The full length hCD164(E1–6) isoform is predicted to contain 9 N-linked and 32 O-linked glycans, and a glycosaminoglycan (GAG) attachment site at the end of E5 and beginning of E6^3,5,13–17^. E1 encodes the first mucin domain, E2 and E3 encode the cysteine rich non-mucin domain, E4 to part of E6 encode the second mucin domain, and the remainder of E6 the transmembrane region, cytoplasmic domain containing a YHTL endocytic motif and 3′UTR. A cytokine binding pocket (reminiscent of TpoR, IL-4R, IL-6R, EpoR, G-CSFR, and GM-CSFR) is located in its non-mucin domain (Fig. 1a). Figure 1b shows a diagrammatic representation of hCD164 and showing putative intra-molecular disulphide bridges between four cysteines in the non-mucin domain^3,5,13–17^. A total of eight cysteines are present and the remaining four may also contribute to intra-molecular or inter-molecular disulphide bridges. The hCD164 glycoprotein has a molecular mass of 80–100 kD under reducing conditions and, under non-reducing conditions, can exist as a homodimer of 160–180 kD. A GAG attachment at the E5–E6 junction (TSGT), association with other components (e.g., the cytoskeleton, growth factors) or the existence of an hCD164 tetrameric form may increase the molecular weight to >220 kD^3,5,13–17^.

## hCD164 monoclonal antibodies and epitopes

Nine Mabs (of varying isotypes; Table 1) were initially generated to hCD164^2,3,5,13–17,23^. While our initial studies defined the epitope reactivity of four Mabs, 103B2/9E10, 105A5, N6B6, and 67D2 with the hCD164 sialomucin, our further studies identified an additional five hCD164 Mabs, 96.1H5, 96.2D2, 96.3F5, 96.10H10, and 96.12H11, which had been produced against hCD34+ cells^3,17^. Cross‐competition experiments between these different hCD164 Mabs indicated that the 105A5 or 103B2/9E10 Mabs were not in hibited in their binding to the hCD164+ hHSPC line KG1A by each other nor by any of the other hCD164 Mabs. Conversely, the N6B6, 67D2, 96.1H5, 96.2D2, 96.3F5, 96.10H10, and 96.12H11 Mabs all cross competed with each other suggesting that they recognise very similar or identical epitopes^17^.

**Table 1:** The characteristics of nine hCD164 monoclonal antibodies.

Monoclonal antibody	Class/Domain (Exon) interaction	Isotype	Molecular weight species of hCD164 in protein lysates	Cross competing (Partial/Complete)	Epitope dependency
105A5	Class I (E1)	mIgM	80–100 kD (monomer) 160–180 kD (homodimer)	No	Long chain sialylated O-linked glycans
103B2/9E10	Class II (E1)	mIgG3	80–100 kD (monomer) 160–180 kD (homodimer)	No	N-linked and O-linked glycans
N6B6	Class IIIA (E2–3)	mIgG2a	80–100 kD (monomer) 160–180 kD (homodimer)	Yes, with Class IIIA and IIIB	Disulphide bridge; conformation
96.1H5	Class IIIA (E2–3)	mIgG1	80–100 kD (monomer) 160–180 kD (homodimer)	Yes, with Class IIIA and IIIB	Disulphide bridge; conformation
96.10H10	Class IIIA (E2–3)	mIgG1	80–100 kD (monomer) 160–180 kD (homodimer)	Yes, with Class IIIA and IIIB	Disulphide bridge; conformation
67D2	Class IIIB (E2–3)	mIgG1	80-100 kD (monomer) 160–180 kD (homodimer) >220 kD	Yes, with Class IIIA and IIIB	Disulphide bridge; conformation
96.12H11	Class IIIB (E2–3)	mIgG1	80–100 kD (monomer) 160–180 kD (homodimer) >220 kD	Yes, with Class IIIA and IIIB	Disulphide bridge; conformation
96.3F5	Class IIIB (E2–3)	mIgG1	80–100 kD (monomer) 160–180 kD (homodimer) >220 kD	Yes, with Class IIIA and IIIB	Disulphide bridge; conformation
96.2D2	Class IIIB (E2–3)	mIgG2b	80–100 kD (monomer) 160–180 kD (homodimer) >220 kD	Yes, with Class IIIA and IIIB	Disulphide bridge; conformation

Further, we demonstrated that hCD164 resembled a subgroup of sialomucins, which include the hHSPC biomarker CD34. While this heavily glycosylated protein subset, like other sialomucins, is rich in serine and threonine residues, it is encoded by multiple exons^2,3,5,13–17,32^. Earlier studies had also demonstrated that CD34 Mabs could be grouped into three Classes based on the resistance or susceptibility of their cognate epitopes to cleavage by the enzymes neuraminidase, chymopapain, and a glycoprotease from *Pasteurella haemolytica*^33–35^. For example, the CD34 epitope recognised by the My10 Class I CD34 Mab was sensitive to both *C. perfringens* sialidase and O*-*sialoglycoprotease treatments; the CD34 epitope identified by the QBEND 10 Class II Mab was sensitive to *O-*sialoglycoprotease, but not to *C. perfringens* sialidase; the CD34 epitope defined by the Tük3 Class III Mab was insensitive to both *C. perfringens* sialidase and O*-*sialoglycoprotease enzymes^33–35^.

Given this, we examined the glycosidase sensitivities of the cell surface hCD164 sialomucin and subsequently of recombinant soluble chimaeric domain-truncation hCD164-Fc/hCD164-His-tagged constructs^13,14,16,17^. We also analysed the global glycosylation patterns (lectin binding, HPLC, and mass spectrometry) of chimaeric recombinant domain-truncation constructs^17^. These latter studies revealed that hCD164 contains sialic acid moieties in both α2-3 and α2-6 linkages on O-glycans and N-glycans, but with O-glycans having a much higher degree of sialylation. Sialyl LewisX (sLeX) was not detected^17^. The N-glycans are more complex than the O-glycans, with high mannose, hybrid and mono-antennary, di-antennary, tri-antennary, and tetra-antennary complex forms (with or without bisecting GlcNAC). Most O-glycans are small core 1 or 2. Predominantly large neutral tri-antennary and tetra-antennary structures occur on N-glycans in mucin domain II, while smaller, bi-antennary structures are present in mucin domain I^17^.

Using a set of nine soluble hCD164 domain truncation mutants comprising different exons (E) but lacking the transmembrane region, namely hCD164E1‐Fc, hCD164E1‐2‐Fc, hCD164E1‐3‐Fc, hCD164E1‐4‐Fc, hCD164E1‐5‐Fc, hCD164EΔ5‐Fc, hCD164E1‐6′‐Fc, hCD164E1,2,4‐Fc, and hCD164E1,3,4‐Fc, we demonstrated that the 103B2/9E10 and 105A5 Mabs recognised all nine soluble proteins, indicating that they react with the region encoded by exon 1^15–17^. The N6B6, 67D2 and 96 series Mabs reacted with the hCD164E1‐3‐Fc, hCD164E1‐4‐Fc, hCD164E1‐5‐Fc, hCD164E1‐6′‐Fc and hCD164EΔ5‐Fc proteins, but not the hCD164E1‐Fc, hCD164E1‐2‐Fc, hCD164E1,2,4‐Fc, and hCD164E1,3,4‐Fc constructs, demonstrating that they require at least exons 2 and 3 for epitope recognition^15–17^. Notably however, none of these Mabs distinguish between the four known hCD164 splice variants^15–17^.

**Fig. 2:**
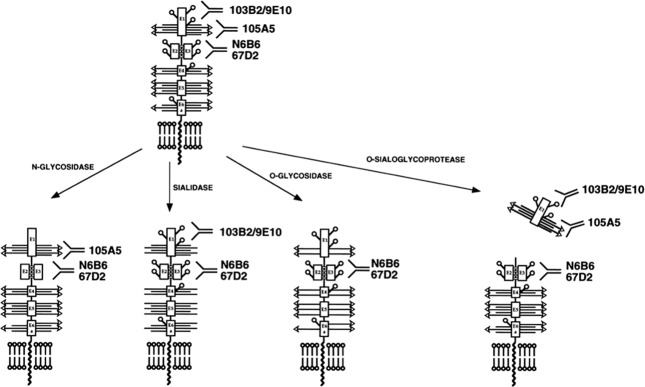
Defining hCD164 epitopes by glycosidase treatments. Schematic representation of hCD164 epitopes deduced from different glycosidase treatments of the hCD164 molecule and the binding of representative Class I (105A5), Class II (103B2/9E10), and Class III (N6B6, 67D2) hCD164 Mabs. □, exon encoded domains; ○, potential N-linked carbohydrates; horizontal bars with or without arrows, potential O-linked carbohydrates; arrows, potential sialic acid motifs on O-linked carbohydrates. (Originally published in *The Journal of Immunology*. Doyonnas R, Yi-Hsin Chan J, Butler LH, Rappold I, Lee-Prudhoe JE, Zannettino AC, Simmons PJ, Bühring HJ, Levesque JP, Watt SM. 2000. CD164 monoclonal antibodies that block hemopoietic progenitor cell adhesion and proliferation interact with the first mucin domain of the CD164 receptor. *J. Immunol*. **165**, 840–851. Copyright © 2000.The American Association of Immunologists, Inc.).

Deglycosylation experiments followed by analyses with hCD164 Mabs were carried out on the hCD164(E1–3)-Fc soluble protein and hCD164 purified from KG1A cells using *N-*glycanase, *O-*glycosidase, sialidase, α-fucosidase, and O*-*sialoglycoprotease treatments, either separately or together (Fig. 2)^13–17^. The epitope recognised by the 105A5 Mab was sialic acid dependent, with sialic acid moieties situated on O*-*glycosylated chains attached to the exon 1-encoded peptide, but not on *N-*linked oligosaccharides. O-glycosidase and O*-*sialoglycoprotease significantly reduced 103B2/9E10 and 105A5 Mab binding to hCD164, but this was not seen with the other hCD164 Mabs. The hCD164 epitope recognised by the103B2/9E10 Mab was sensitive to *N-*glycanase treatment, and hence is dependent on the *N-*linked carbohydrates of exon. In contrast, the epitopes recognised by the remaining hCD164 MAbs were not removed by the deglycosylation procedures used. Thus, by comparing hCD164 Mabs with the CD34 Mab Classes, the hCD164 Mabs could be classified into three analogous categories^13–17^. Hence, the epitope recognised by the Class I 105A5 Mab is present on hCD164 mucin domain I and is associated with long chain sialylated O-linked glycans. The epitope recognised by the Class II 103B2/9E10 Mab is also present on mucin domain I and is dependent on N-linked and O-linked glycans, but independent of sialylation. The remaining seven Class III Mabs react with conformation-dependent epitopes that require the co-expression of the cysteine-rich domain, encoded by E2 and E3, which lies between mucin domains I and II. Their binding relies on intramolecular disulphide bridges, which are resistant to glycosidase cleavage and principally encompass the hCD164 peptide backbone^13,14,16,17^. The Class III Mabs are further divided into closely associated Class IIIA and IIIB Mabs, with the seven Mabs partially or completely cross-competing with each other^13,14,16,17^. Class IIIA Mabs (N6B6, 96.1H5, and 96.10H10), like their Class I and II counterparts, identify hCD164 monomers or homodimers, while the Class IIIB antibodies (67D2, 96.12H11, 96.3F5, and 96.2D2) also bind to a > 220 kD hCD164 protein species^13,14,16,17^. As indicated earlier, the latter may be an hCD164 tetramer, a hCD164 monomer or homodimer in association with other, possibly cytoskeletal or growth factor, components, or a GAG containing hCD164 species, an issue not yet resolved (Fig. 1)^3,5,13–17^.

## Cellular distribution of hCD164 epitopes

Assessment of the cellular distribution of Class I and II hCD164 epitopes^2,3,5,23,26,31^ revealed their expression on CD45^+^CD34^+^ hHSPCs throughout ontogeny. Notably, they are present on CD34^+^ clusters located on the ventral floor of the dorsal aorta in the week 5–6 developing human embryo, and on a proportion of CD34^+^ cells from foetal liver, cord blood, bone marrow, and mobilised peripheral blood^23^. Furthermore, Lin^neg^CD34^lo/neg^ or CD34^int+extneg^CD38^lo/neg^, CD133^hi^CD34^hi^CD38^lo/neg^, and CD133^hi^CD34^lo/neg^ repopulating HSPCs express abundant hCD164, of which the CD34^+^CD164^hi^ HSPCs expand more in ex vivo cultures than bulk CD34^+^ HSPCs^3,5,6,19–22,28,29,31^. Recent studies by Pellin’s group used the Class IIIB 67D2 Mab to demonstrate that CD34^+^CD164^hi^, but not CD34^+^CD164^lo^, cells sustained early and late in vivo human haematopoietic reconstitution in NBSGW immunodeficient mice^6^. Importantly, hCD164 is also a biomarker for hMSCs/hSSCs, including CD271^hi^CD45^lo/neg^ and CD56^+^ or CD56^neg^ primary bone marrow hMSC^7,8,25,26^. Notably, the Class IIIB Mab (67D2) has been used to identify and isolate PDPN^+^CD146^neg^ CD73^+^CD164^+^ self-renewing, serially transplantable hSSCs with multipotent (osteogenic, chondrogenic, and haematopoietic supporting stromal) potential from the human bone growth plate and diaphyseal zones^7^. Significantly, these hSSCs exist at the apex of the human skeletogenic differentiation hierarchy, are present in human foetal and adult bones, can be generated from BMP-2 stimulated human adipose MSCs and iPSCs, expand locally following acute skeletal injury and maintain human haematopoiesis^7,8,11^.

While all the hCD164 epitopes described to date are present on hCD34^+^ and hCD133^+^ hHSPC subsets and on hBM MSCs, this is in direct contrast to their differential expression in other postnatal haematopoietic and non-haematopoietic tissues^2,3,5,13–17,23^. We observed that the epitopes detected by the Class I and II Mabs can be differentially expressed and distributed on reciprocal cell subsets in some tissues, while the Class III Mabs stained cells that bound both or either of the Class I and/or II hCD164 Mabs^23^. For example, the epitope recognised by the 103B2/9E10 Class II Mab, but not that by the 105A5 Class I Mab, is present on most vascular endothelium, on some high endothelial venules in lymphoid tissues, on the venous sinuses in spleen, on the thymic subcapsular epithelia which is associated with pre-T lymphoid cell trafficking and on the basal-layer epithelia of the tonsil and skin. In contrast, the epitope recognised by the 105A5 Class I Mab is present on lymphoid cells in tissues that are involved in lymphoid/blood cell recirculation, such as in the tonsil, spleen, and gut^23^. However, the CD1^+^ and cortical and medullary CD43^+^ thymic lymphoid cells stain weakly with the Class I, but not at all with the Class II, hCD164 Mabs, while thymic macrophages express both the hCD164 epitopes defined by the Class I and II Mabs^23^.

## The role of hCD164 in regulating cell fate

The epitopes defined by the different Classes of hCD164 Mabs are implicated in possessing functional roles in haematopoiesis as illustrated by in vitro studies or based on hCD164 structural studies. Engaging the hCD164 molecule with specific hCD164 Mabs can modulate CD34^+^ or CD133+hHSPC proliferation, differentiation, adhesion to, and retention or migration in different microenvironmental niches. As a first exemplar, the hCD164 103B2/9E10 Class II Mab-defined epitope partially blocks adhesion of CD34^+^ and CD133^+^ hHSPCs to bone marrow MSCs^3,5,13,14,31^. Ligating this epitope with the 103B2/9E10 Class II Mab also inhibits cytokine-driven (IL-3, IL-6, SCF, and G-CSF) recruitment of single CD34^+^CD38^lo/neg^ hHSPCs into the cell cycle in vitro^3,5,13,14^. Such effects are indicative of cognate ligands for hCD164 acting in cis or trans, albeit not yet fully defined. Our findings show that hCD164 lacks sLeX moieties required for P-selectin or E-selectin binding, while its sialylation in the absence of fucosylation suggests that hCD164 is a potential Siglec ligand, with preliminary studies indicating hSiglec-5 (but not hSiglec-3, hSiglec-7, hSiglec-8, hSiglec-9, or hSiglec-10) binding^13–15,17^. The second example demonstrates that culturing hBM CD34^+^ cells with IL-1β, IL-3, IL-6, G-CSF, GM-CSF, and SCF for 21 days in the presence of the Class I 105A5 or Class II 103B2/9E10 hCD164 Mabs reduces the absolute number of both total nucleated cells generated, and, when erythropoietin is added as well, decreases the development of clonogenic BFU-E and CFU-GM^13–15^. Furthermore, hCD164 is a key component of the CXCR4-VLA-4-VLA-5 complex, regulating CD133^+^ hHSPC CXCL12-mediated migration or retention in the hHSC niche and promoting CXCR4-mediated signalling, a process inhibited by the 103B2/9E10 Class II hCD164 Mab^31^. Although functional effects of engaging the hCD164 epitopes with Class III Mabs have not been fully assessed, the full length hCD164 contains a potential cytokine binding pocket located in its non-mucin domain, as well as a potential GAG attachment site at the E5-E6 junction of hCD164^2,3,5,13–17^, that could act in concert to modulate cytokine-mediated, chemokine-mediated, or adhesion-mediated effects on the same or opposing cells. While this is purely hypothelical, it is important to keep in mind that these and other Mabs chosen to isolate hHSCs might inadvertently affect the function or fate of the hHSCs when assessed in vitro or in vivo.

## Biomarkers and lineage hierarchies

Biomarkers that identify definitive, but rare, repopulating human bone marrow stem cells have been the “holy grail” of stem cell research since the seminal discovery of monoclonal antibodies by Koehler and Milstein over 5 decades ago^36^. These, when coupled with the increasingly sophisticated technological developments^37–44^ of cell sorting, surrogate in vivo models, gene editing, single cell barcoding, single cell deep sequencing, lineage tracing and fate mapping in vitro and in vivo, and human transplantation or regenerative medicine, have fuelled a series of debates on the hierarchical relationships of these human stem cells with their immediate progeny, and on selecting the best biomarkers and cell subsets for therapeutic use. To this end, biomarkers, lineage trees and lineage relationships have been constantly changing with progressive technological developments and research. Examples of these are indicated in the attached references^6,7,41–62^.

Deciphering lineage commitment processes with biomarkers is critical to our understanding of human haematopoiesis^3,7,8,30,37,55–62^, and must not be hindered by the use of reagents that modulate hHSPC function in vivo. Hypotheses are hotly debated; the classical model describes a structured hierarchy of lineage commitment, with early branching of lymphoid and erythro-myeloid lineages. Other hypotheses include lineage-biased HSCs, early megakaryocyte lineage branching followed by erythroid, myeloid, and lymphoid lineage transitions, early branching of erythroid-megakaryocyte precursors from lymphoid–myeloid precursors, and a continuum of low-primed hHSPCs from which unilineage cells differentiate (CLOUD-HSPCs)^41,42,44–55,59,61,62^. Pellin et al.^6^ have stated that hCD164 can ‘preserve the resolution of the single-cell events of the more-primitive compartments, whereas at the same time maintaining a full representation of the late cell fate branching’, leading them to propose a modification of the classical hierarchical model, in which ‘human hematopoiesis develops along early cell fate bifurcations occurring in a continuum of states forming a hierarchical-like structure’ with early priming to either lymphoid/dendritic cell/monocyte/granulocyte or erythroid/megakaryocyte /basophil commitment.

When defining lineage hierarchies and developing new cellular products with hCD164 as a biomarker, it is important to remember that, while Pellin et al.^6^ and Chan et al.^7^ used the 67D2 Class IIIB hCD164 antibody to identify hHSCs and hSSCs, different classes of hCD164 antibodies possess different avidities for this molecule and, by interfering with ligand binding sites, these may alter human hHSPC and hSSC function and hence fate. The information garnered from previous research should be carefully reviewed when considering the choice of hCD164 antibodies in future clinical studies. This said, Class II, and to a lesser extent, Class I hCD164 antibodies, with isotypes distinct from the Class III antibodies, may prove beneficial for confirming the purity of and hence establishing release criteria for the hCD164 Class III-enriched hHSC and hSSC subsets for in vivo therapy.

In conclusion, using this knowledge in association with new cellular therapies will undoubtedly contribute to successful treatments for life threatening and chronic diseases, which are substantial burdens on both patient quality of life and healthcare systems globally^63,64^.
